# Data handling on a sequential multichannel analyser computerized (SMAC): using a low-cost minicomputer

**DOI:** 10.1155/S1463924682000091

**Published:** 1982

**Authors:** R. Dugdale, S. Harrison, D. M. Robertshaw

**Affiliations:** Biochemistry Department Bradford Royal Infirmary Duckworth Lane West Yorkshire Bradford BD9 6RJ UK


Journal of Automatic Chemistry, Volume 4, Number 1 (January–March 1982), pages 32–34

### Data handling on a sequential multi-channel analyser computerized (SMAC): using a low-cost minicomputer

**R. Dugdale, S. Harrison and D. M. Robertshaw**

*Biochemistry Department, Bradford Royal Infirmary, Duckworth Lane, Bradford, West Yorkshire BD9 6RJ, UK*

### Introduction

Since the introduction of automatic analysers into clinical chemistry laboratories, the trend has been towards using computers to help cope with the increased data-load. Many laboratories have approached this need to handle large amounts of information by using computers to organize the laboratory's work from start to finish, i.e. from receipt of specimen to production of a final report quite often via work-sheet production, cumulative report production and patient data storage. Systems such as Phoenix (produced by Computer Technology Ltd, Hemel Hempstead, Hertfordshire, UK) and Laboratory Data Manager (Technicon Instruments Corporation, Tarrytown, New York 10591, USA) are examples of computerization intended to help in all areas of the laboratory.

Laboratory computer systems can, however, impose a certain rigidity because once the system is operational the department can only be run one way, and in conjunction with the computer. By streamlining and simplifying the receipt, separation and reporting schemes in the authors' laboratory, it was possible to handle large work-loads (over 150 000 requests per year) in conjunction with mechanized analysis, without investing large sums of money (sometimes over £30 000) in hardware.

Two areas where, because of their speed, computers can be a definite advantage are data storage and the mathematical calculation of statistics associated with quality assurance (QA).

The introduction of a sequential multi-channel analyser, computerized (SMAC) into the laboratory at Bradford Royal Infirmary and the availability of relatively cheap (under £2000) minicomputers, such as PET (manufactured by Commodore Business Machines Inc., 901 California Avenue, Palo Alto, California, USA) and Sorcerer (Exidy Inc., 390 Java Drive, Sunnyvale, California, USA), which can easily be interfaced to the SMAC output, prompted the authors to try to use a minicomputer to ease data storage and the QA aspects of the profiling service.

Before the computer system was available the quality of results from the SMAC was assessed on a regular basis by the operators, transcribing the results of the QA specimen (at every eighth position) onto a stencilled sheet. This forced the operators to examine these answers every eight specimens and any deviation from pre-set values enabled the problem to be corrected and patient results repeated. If any delay occurred either in reporting results or in examining QA data many samples might have been analysed before the problem was noticed. This could lead to many more specimens having to be repeated than was really necessary. At the end of each day, statistics were applied to the results on the QA sheets to observe any long-term trends and change in precision.

Data storage from the SMAC consisted of the Laboratory Information System's (L.I.S.) output linked to a fast line-printer and because of the simplified form in which the L.I.S. outputs the data, 33 full patient profiles could be kept on one sheet of fan-fold paper measuring $14\frac{1}{2}$ in. by 11 in. Patient results were reported by sticking peel-off labels printed on the SMAC printer onto the original request form after matching I.D. numbers.

The object of this work was to utilize a minicomputer interfaced to the L.I.S. output, to provide a continuous statistical view of QA specimens and store these and all patient data on a floppy disc for further processing. The intended advantages of this initial stage were to remove the need for manual statistical analysis of the QA results at the end of each day by having the computer do this, and also eliminate the need for the SMAC operators to scrutinize every control as it was produced, since an alarm would activate if any value fell outside certain criteria.

By storing all patient data on floppy disc (2000 patient profiles can be held on one disc), the selected populations can be recalled or patients selected provided these were coded. This would have the advantage of enabling biochemical changes with respect to age, pregnancy, hospitalization etc., to be studied.

### Method

The minicomputer chosen for this application was the Exidy Sorcerer with 32 kilobytes of random access memory and twin floppy-disc unit, providing a further 630 kbytes of storage (see figure 1). No extra hardware interface was required between the analyser and computer since the RS232 serial data-stream from the SMAC could be connected directly into the system. A normal television set was modified to act as a visual display unit (VDU).

Two major programs were written to meet the software requirements. The first program performed real-time acquisition, storage and display of all incoming data. The control samples (two levels) were numerically coded for easy identification and these were processed further. This involved checking the individual parameter values to determine whether or not they fell within appropriate limits. Any value falling outside the limits sounds an alarm which has to be manually cancelled. Running means and standard deviations are then determined for each parameter and displayed on the screen (see figure 2). A considerable portion of the software for string handling and input of data was written in the form of BASIC-callable machine code subroutines. This greatly increased the programme speed and enabled the system to work effectively in the real-time mode.

The second program was a review program, using the day's control data which could be selectively edited to give overall means and standard deviations for trend analysis. In addition it was possible to recall any patient data as necessary.

Several subsidiary programs have also been developed. These enable numerical listing and reformating of patient results, control value limits to be changed, and the controls to be

 0142-0453/82/0401-0032 $05.00 © 1982 Taylor & Francis Ltd

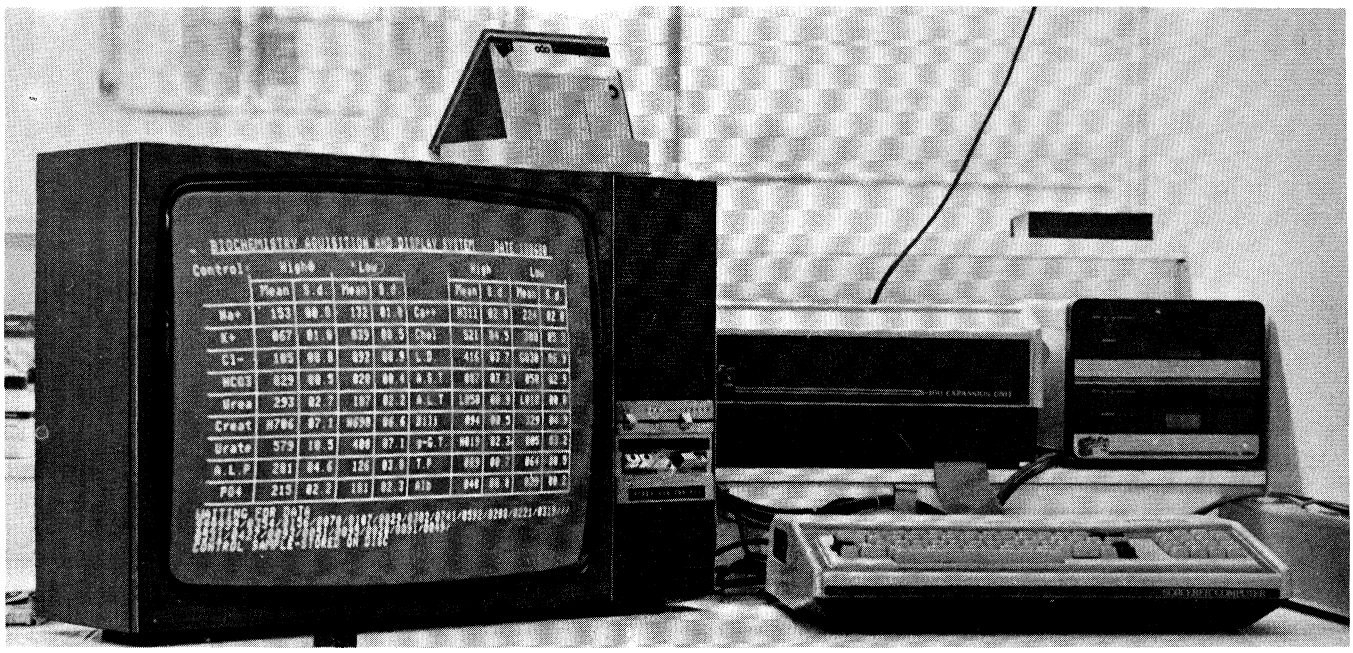

*Figure 1. The Exidy Sorcerer minicomputer used in this work.*

realistically evaluated after the run.

When the SMAC is being run the operators use the system as follows. If the alarm sounds it indicates that either one or more values for a control is outside ± 3 SD of a previously established *mean* value. In these cases the computer does not include the offending result(s) in the calculations, but places an 'L' or 'H' next to the appropriate variable (figure 2) so that the SMAC operators can assess the situation. The result responsible for the alarm is noted and appropriate action taken: either turning the instrument off to correct the fault and repeating the patient analysis, or accepting the result if the problem is caused by a bad peak (asterisk) or carry-over.

At the end of each day (or run) the QA data is reviewed. Firstly the entire day's control values are printed out from the disc to give hard copy of each set of controls. The program then asks the operator if any controls need excluding from the overall daily mean, SD and coefficient of variance (CV). Any control in a batch of results which had been repeated because of bad QA or any parameter which has not been reported on patients can then be eliminated from the final QA assessment. The final mean, the SD and CV of each day are therefore representative of the results actually reported on patients.

Once the computer has calculated the statistical parameters these are printed-out on the line-printer (figure 3) for plotting and day-to-day evaluation. Then the computer runs the sort program, which produces a numerical listing of patient's results and also reformats the raw data from the L.I.S. by adding decimal points and removing leading zeros. This listing means that repeats/dilutions etc. for a particular patient are all listed together, and if it is necessary to use this hard copy to check

*Biochemistry aquisition and display system Date: 180680*

| Control | High | | Low | | | High | | Low | |
|---|---|---|---|---|---|---|---|---|---|
| | Mean | S.D. | Mean | S.D. | | Mean | S.D. | Mean | S.D. |
| Na+ | 153 | 00·4 | 132 | 01·0 | Ca++ | 311 | 02·0 | 224 | 02·0 |
| K+ | 067 | 00·8 | 039 | 00·5 | Chol | 521 | 04·1 | 308 | 05·3 |
| Cl− | 105 | 00·8 | 092 | 00·9 | L.D. | 416 | 03·0 | G830 | 06·9 |
| HCO3 | 029 | 00·5 | 020 | 00·4 | A.S.T. | 086 | 02·9 | 050 | 02·9 |
| Urea | 293 | 01·8 | 187 | 02·2 | A.L.T. | L058 | 00·9 | L010 | 00·0 |
| Creat | 706 | 07·1 | H698 | 06·6 | Bili | 093 | 00·3 | 329 | 04·9 |
| Urate | 578 | 10·3 | 408 | 07·1 | g-G.T. | 019 | 02·3 | 005 | 03·2 |
| A.L.P. | 280 | 04·5 | 126 | 03·8 | T.P. | 089 | 00·7 | 064 | 00·5 |
| PO4 | 215 | 01·8 | 181 | 02·3 | Alb | 047 | 00·8 | 029 | 00·2 |

*Figure 2. Cumulative QA statistics and SMAC output data on a modified television screen used as a VDU (from print-out).*

patient results they can be found more easily.

### Discussion

At the present time the system is only being used for QA assessment and data storage, but there is considerable potential still to be released. Several immediate advantages are already apparent. The very labour-intensive process of statistics calculation is eliminated—these are done quickly at the end of the day, not the following day. This alone probably saves 10 hours of technician time each week.

The SMAC operators are now able to concentrate on the running of the SMAC and reporting results, knowing that any QA problem will be drawn to their attention. Since the variable at fault is also indicated, no time is wasted checking all the values on each QA specimen. Because of the review program, gradual day-to-day changes in the mean and SD can be observed quickly and easily and the appropriate action taken before the next run, rather than a full day later because of delay in statistics calculation.

Because the system is vetting the QA specimens continuously no delay in reporting results occurs. The QA specimens are checked as they arise, not in batches at the end of the run or end of the day. This means that any suspect results can be repeated immediately and the delay in getting the results back to the wards is minimized.

The system has been introduced without any alterations to the reception, separation, analysis and reporting aspects of the laboratory. This might not have been the case had a large laboratory computer been introduced.

Further, no extra clerical or technical staff are required to operate the SMAC and the computer is both quick and simple to use.

The future potential of the system is in merging patient details with the L.I.S. output, which would allow patient data to be recalled from disc by name or hospital number. Programs are also being developed for computer plotting of day-to-day QA and also an editing program to correct results for dilutions and checks.

Overall, the minicomputer can provide rapid statistical analysis of QA specimen results, patient storage and recall and population-data evaluation. It is not expensive in terms of capital cost, nor does it impose any limitations on laboratory organization. It is an addition to the laboratory, not a replacement, and yet can be developed to suit future needs.

CONTROL STATISTICS FOR 010780

| | HIGH-CONTROL | | | LOW-CONTROL | | |
|---|---|---|---|---|---|---|
| | Mean | S.D. | C.V. | Mean | S.D. | C.V. |
| Na+ | 155·66 | 2·23 | 1·43 | 133·31 | 1·41 | 1·04 |
| K+ | 6·84 | 0·135 | 1·97 | 3·91 | 0·036 | 0·93 |
| Cl− | 106·29 | 1·13 | 1·06 | 91·73 | 1·16 | 1·20 |
| HCO3 | 28·95 | 0·88 | 3·06 | 19·89 | 0·64 | 3·21 |
| Urea | 30·12 | 0·444 | 1·47 | 18·93 | 0·377 | 1·99 |
| Creat | 717·79 | 15·59 | 2·17 | 689·15 | 13·48 | 1·95 |
| Urate | 596·12 | 9·69 | 1·62 | 408·00 | 6·58 | 1·61 |
| ALP | 297·16 | 4·84 | 1·63 | 128·00 | 4·55 | 3·55 |
| PO4 | 2·1804 | 0·0285 | 1·30 | 1·8168 | 0·0334 | 1·83 |
| Ca+ | 3·1329 | 0·0538 | 1·72 | 2·8723 | 0·0403 | 1·76 |
| Chol | 5·3587 | 0·0831 | 1·55 | 3·0936 | 0·018 | 3·29 |
| L.D. | 425·45 | 10·81 | 2·54 | 824·94 | 15·32 | 1·85 |
| A.B.T. | 92·84 | 3·19 | 3·44 | 54·50 | 4·43 | 8·12 |
| A.L.T. | 70·08 | 3·04 | 4·33 | | | |
| Bili | 99·54 | 2·19 | 2·20 | 344·68 | 9·07 | 2·63 |
| p-G.T. | 24·50 | 2·43 | 9·92 | | | |
| T.P. | 90·20 | 1·25 | 1·39 | 63·57 | 1·04 | 1·63 |
| Alb | 48·73 | 0·79 | 1·65 | 29·42 | 0·59 | 2·00 |

*Figure 3. Printed output from the Sorcerer at the end of a run, after editing the QA values.*

### Conference announcement

*1982 Pittsburgh Conference and Exposition on Analytical Chemistry and Applied Spectroscopy*

The 33rd Pittsburgh Conference will be held from 8 to 13 March 1982 at the Atlantic City Convention Hall in New Jersey, USA.

The Conference's Technical Programme will run from 8 to 12 March, the Exposition from 8 to 11 March and the Special Short Course (on the use of infra-red group frequencies) from 12 to 13 March.

There will be 90 technical sessions, 850 papers will be given and there are 17 invited symposia.

520 companies dealing with spectroscopic and general analytical instrumentation equipment have booked booths for the Exposition.

The organizers have announced dates for future Pittsburgh Conferences: the 34th will be held from 7–11 March 1983 again at Atlantic City; the 35th is undecided; the 36th will run from 25 February to 1 March 1985 at New Orleans; the 37th will be held from 17 to 21 March 1986 at Atlanta, Georgia.

*Further information from The Pittsburgh Conference, 437 Donald Road, Department J-212, Pittsburgh, PA 15235, USA.*

